# Differential protective effects of Family Income-to-Poverty-Ratio on electronic cigarette, depression, and obesity of Black and White Americans

**DOI:** 10.3934/publichealth.2024060

**Published:** 2024-12-11

**Authors:** Shervin Assari

**Affiliations:** Departments of Urban Public Health, Internal Medicine, and Family Medicine, Charles R. Drew University of Medicine and Science, Los Angeles, CA, USA

**Keywords:** social determinants, ethnic groups, health equity, Family Income-to-Poverty-Ratio, Black/White Americans

## Abstract

**Background:**

The Family Income-to-Poverty-Ratio (FIPR) is a recognized indicator of socioeconomic status, and influences a wide range of health and behavioral outcomes. Yet, marginalized and racialized groups, particularly Black individuals, may not reap comparable health benefits from their socioeconomic advancements as their non-Hispanic, White counterparts. This discrepancy is indicative of a phenomenon known as the minorities' diminished returns.

**Aims:**

This study investigates the differential impact of the FIPR on depression, obesity, tobacco use, and e-cigarette use between Black and White adults.

**Methods:**

Using data from the 2022 National Health Interview Survey (NHIS), which included 21,354 non-Hispanic adults from both White and Black racial groups, this research employed structural equation modeling to assess the relationship between the FIPR and health outcomes, including depression, obesity, and e-cigarette use.

**Results:**

The analysis identified significant interactions between FIPR and race across all the examined outcomes. Contrary to expectations, the findings suggest that the protective effects of higher income levels on health and healthy behaviors are less pronounced for Black individuals compared to White individuals.

**Conclusion:**

The study underscores the substantial societal and environmental barriers that hinder Black families and individuals from converting their FIPR and socioeconomic resources into concrete health benefits, such as an enhanced mental and physical well-being. To redress these racial health disparities, targeted interventions are crucial, particularly those that focus on bridging the employment and marriage rate gaps caused by educational disparities among Black communities. A comprehensive approach that extends beyond simple access to education is imperative to eliminate the societal obstacles that limit the socioeconomic benefits for Black populations.

## 1. Introduction

The Family Income-to-Poverty Ratio (FIPR) [1], also referred to as the poverty ratio [2] or income-to-needs ratio [3], comprises a set of economic terms that are pivotal in assessing socioeconomic status (SES). These relative SES indicators are intricately linked with various mental, physical, and behavioral health outcomes [3],[4]. A higher FIPR is consistently associated with an enhanced physical and mental health, as well as a reduction in substance use, including lower tobacco consumption [5]–[8]. This correlation emphasizes SES as a fundamental determinant of health and well-being [9].

However, a review of recent literature on SES indicators such as education, employment, income levels, and marital status unveils a troubling trend: the advantages conferred by these SES assets often disproportionately benefit White individuals over their Black counterparts. Notwithstanding their achievements in education, employment, and marital status, Black individuals are still more likely to encounter significant health risks. This disparity is notably evident in mental health conditions such as depression, physical health issues such as obesity, and behavioral and substance use concerns, including tobacco use. Consequently, high SES Black individuals are susceptible to depression, obesity, substance use, and poor self-rated health (SRH), whereas their White counterparts with similar educational and income levels are largely shielded from these adverse outcomes. This persistent inequality challenges the conventional belief held by policymakers that SES is the primary driver of racial and ethnic health disparities and that bridging the SES gap would mitigate racial health inequities.

The concept of the Minorities' Diminished Returns (MDRs) introduces a critical reevaluation of these disparities, attributing them to systemic racism and social stratification [10]–[13]. These forces engender a context in which high-SES Black individuals face a multitude of societal, environmental, and historical challenges that are deeply embedded in the social fabric and are not readily resolved by merely addressing SES disparities [10]–[13]. Despite higher education, income, and stable employment and marital statuses, high-SES Black families are more likely to live in poorer neighborhoods. Moreover, moving to predominantly White areas often increases their exposure to discrimination and various forms of stress, further limiting their access to economic resources, environmental quality, and healthy choices.

Yet, the application of these insights to the FIPR—a comprehensive SES indicator that considers household size—has been insufficiently explored. This measure adds another dimension to the comparison between racial groups, particularly given the variation in the average household sizes among Black and White populations. While the diminished returns of education have been previously shown, there is a dearth of studies that examined the diminished returns of FIPR. As different SES indicators have different effects [14]–[18], and given that the FIPR is a more proximal SES indicator than education (for most adults, from a temporal aspect, education attainment precedes current FIPR measure), our aim is to compare the returns of the FIPR on health behaviors across Black and White individuals.

In this study, leveraging data from the National Health Interview Survey (NHIS) 2022, we investigate the MDRs of income on depression, obesity, and e-cigarette use. We hypothesize that although the protective effects of income are observable across the population, they are significantly less potent for Black individuals compared to White individuals. This phenomenon underscores the extensive impact of racial disparities within the realm of socioeconomic advantage.

## 2. Materials and methods

### 2.1. Design and setting

The study utilized data from the 2022 National Health Interview Survey (NHIS), which was conducted by the Centers for Disease Control and Prevention (CDC). This survey collected comprehensive data on health status, health behaviors, and socioeconomic status (SES) among the US population.

### 2.2. Sample and sampling

A nationally representative sample of non-institutionalized adults aged 18–99 across all racial/ethnic groups in the US was selected, totaling 27,648 participants. For this analysis, we only included non-Hispanic Black and White individuals; therefore, Native American, Asian, mixed race, other race, and those who did not report their race were excluded. All Latino/Hispanic people were excluded. Our analytical sample was 21,354 non-Hispanic Black or non-Hispanic White people.

### 2.3. Measures

Independent Variable: the FIPR. The NHIS calculated and imputed the income data. Based on the income data, the NHIS calculated a FIPR score ranging from 1 to 14, with a higher score indicating a higher SES.

The measurable outcomes of the study were as follows:

Depression: Based on the patient Health Questionnaire-8, we used severe depression as the outcome (scores 15–25).

Obesity: Based on body mass index of 30 or more, obesity was one of the outcomes;

Electronic Cigarette: Ever use of electronic cigarette was an outcome.

The confounders of the study were as follows:

Age: Measured in years since the individual's last birthday, ranging from 18 to 99, and treated as a continuous variable.

Sex: Coded dichotomously, with 1 indicating male.

Educational attainment: an independent variable, operationalized on an 11-point scale ranging from 0 (Never attended, kindergarten only) to 10 (Professional School or Doctoral degree, e.g., MD, DDS, DVM, JD, PhD, EdD), and treated as continuous.

Marital Status: Distinguished as married versus any other status at the time of the survey.

Employment Status: Categorized as employed versus not employed or not in the labor market at the time of the survey.

Moderator: Race included Black and White (reference group).

### 2.4. Statistical analyses

Data were analyzed using Stata, version 18.0 (StataCorp LLC, College Station, TX). Frequencies and percentages were reported in univariate analyses, while bivariate analyses examined differences between the Black and White populations across all the study variables. A Pearson correlation was employed to evaluate correlations between the variables, with the analyses being stratified by ethnicity. Path modeling, which is a subtype of structural equation modeling (SEM), was applied to the pooled sample. This model incorporated an interaction term between educational attainment and race (Black), targeting depression, obesity, and electronic cigarette use as the outcome variables, and considered marital status and employment as potential mediators. A positive and significant path coefficient for educational attainment with respect to employment and marital status indicated the beneficial effects of education. Conversely, a negative and significant interaction between race and educational attainment on the outcomes suggested that the positive impacts of education on the health outcomes are less pronounced for the Black individuals compared to the White individuals. The results are presented with standardized coefficients (Beta), standard errors (SE), 95% confidence intervals (CIs), and p-values, with the statistical significance set at p ≤ 0.05.

### 2.5. Ethics

The National Center for Health Statistics (NCHS) maintained the Institutional Review Board (IRB) approval from the NCHS Research Ethics Review Board (ERB). Due to the use of secondary, de-identified, publicly available data for this study, IRB oversight was not required.

## 3. Results

From all 21,354 non-Hispanic Black or White participants, 18,242 were White and 3112 were Black. As Table 1 shows, the White individuals were older than the Black individuals. The White participants had a higher education and FIPR (SES) than the Black participants.

**Table 1.** Descriptive data, weighted.

	All (21,354)				White (18,242)				Black (3112)			
	Mean	SE	95%	CI	Mean	SE	95%	CI	Mean	SE	95%	CI
Age*	46.10	0.42	45.28	46.92	50.59	0.20	50.19	50.99	46.10	0.42	45.28	46.92
Education*	5.18	0.05	5.07	5.29	6.03	0.03	5.97	6.08	5.18	0.05	5.07	5.29
FIPR (1–14)*	8.31	0.11	8.09	8.53	10.59	0.05	10.50	10.68	8.31	0.11	8.09	8.53

Note: FIPR: Family Income-to-Poverty-Ratio. * p < 0.05.

As Table 2 shows, the FIPR was inversely associated with being obese, being depressed, and using e-cigarettes. At the same time, the FIPR was positively correlated with education, employment, and being married. Being Black was inversely associated with the FIPR and education.

**Table 2.** Bivariate correlations.

	Black	education	FIPR	Employed	Married	Age	Male	Depressed	Obese	E-Cig
Black	1.00									
Education	−0.14*	1.00								
FIPR	−0.21*	0.46*	1.00							
Employed	0.02	0.22*	0.29*	1.00						
Married	−0.16*	0.16*	0.31*	0.06	1.00					
Age	−0.08*	−0.07*	−0.02	−0.52*	0.05	1.00				
Male	−0.04*	−0.01	0.08*	0.10*	0.07*	−0.05	1.00			
Depression	−0.01	−0.07*	−0.12*	−0.06*	−0.07*	−0.04	−0.04	1.00		
Obese	0.09*	−0.10*	−0.08*	0.02	−0.01	−0.02	−0.01	0.05	1.00	
E-Cig	−0.04*	−0.08*	−0.08*	0.12*	−0.12*	−0.32*	0.06	0.11*	0.00	1.00

Note: FIPR: Family Income-to-Poverty-Ratio. * p < 0.05.

**Figure 1.**
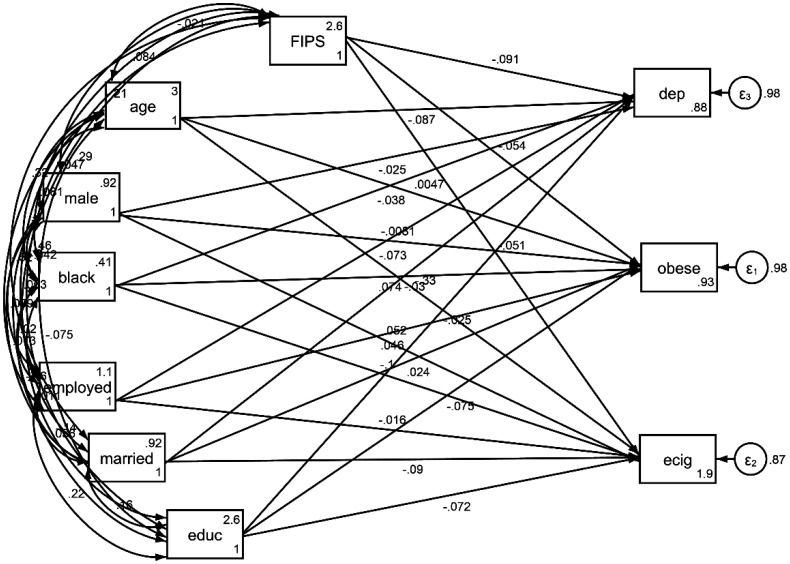
Path model 1 (structural equation model) on the association between incarceration before age 50 and self-rated health at retirement.

As Table 3 and Figure 1 show, a higher FIPR was associated with a lower risk of obesity, e-cigarrete use, and depression.

**Table 3.** Summary of path model 1 (structural equation model) on the association between Family Income to Poverty Ratio (FIPR), race, and health outcomes.

		Beta	SE	95%	CI	p
Obese						
	Age	0.005	0.008	−0.011	0.021	0.564
	Employed	0.046	0.009	0.029	0.063	<0.001
	Black	0.074	0.007	0.060	0.088	<0.001
	Male	−0.008	0.007	−0.022	0.005	0.243
	Education	−0.075	0.008	−0.090	−0.059	<0.001
	FIPR	−0.054	0.008	−0.070	−0.037	<0.001
	Married	0.024	0.007	0.009	0.038	0.001
	Intercept	0.933	0.036	0.861	1.004	<0.001
E-Cig						
	Age	−0.332	0.007	−0.346	−0.317	<0.001
	Employed	−0.016	0.008	−0.032	0.000	0.043
	Black	−0.102	0.007	−0.115	−0.088	<0.001
	Male	0.052	0.007	0.039	0.064	<0.001
	Education	−0.072	0.007	−0.086	−0.057	<0.001
	FIPR	−0.051	0.008	−0.066	−0.035	<0.001
	Married	−0.090	0.007	−0.103	−0.076	<0.001
	Intercept	1.875	0.032	1.812	1.937	<0.001
Depressed						
	Age	−0.087	0.008	−0.103	−0.071	<0.001
	Employed	−0.073	0.009	−0.090	−0.056	<0.001
	Black	−0.038	0.007	−0.052	−0.024	<0.001
	Male	−0.025	0.007	−0.038	−0.011	<0.001
	Education	−0.025	0.008	−0.040	−0.009	0.002
	FIPR	−0.091	0.008	−0.108	−0.075	<0.001
	Married	−0.030	0.007	−0.045	−0.016	<0.001
	Intercept	0.877	0.036	0.807	0.946	<0.001

Note: FIPR: Family Income-to-Poverty-Ratio.

**Figure 2.**
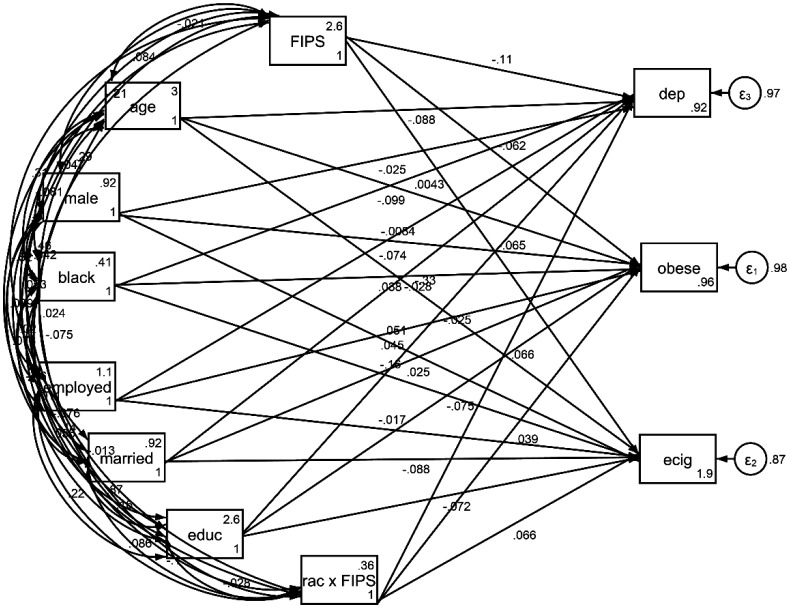
Path model 2 (structural equation model) on the association between incarceration before age 50 and self-rated health at retirement.

As Table 4 and Figure 2 show, although a higher FIPR was associated with a lower risk of obesity, e-cigarette use, and depression, there were significant interactions between race and the FIPR on all these outcomes, suggesting that the effects of the FIPR on obesity, e-cigarette use, and depression were all weaker in the Black individuals compared to the White individuals.

**Table 4.** Summary of path model 2 (structural equation model) on the association between Family Income to Poverty Ratio (FIPR), race, and health outcomes.

		Beta	SE	95%	CI	p
Obese					
	Age	0.004	0.008	−0.012	0.020	0.598
	FIPR x Race (Black)	0.039	0.015	0.009	0.070	0.010
	Employed	0.045	0.009	0.028	0.063	<0.001
	Black	0.038	0.016	0.007	0.069	0.015
	Male	−0.008	0.007	−0.022	0.005	0.224
	Education	−0.075	0.008	−0.090	−0.059	<0.001
	FIPR	−0.062	0.009	−0.080	−0.045	<0.001
	Married	0.025	0.007	0.010	0.039	0.001
	Intercept	0.956	0.037	0.883	1.030	<0.001
						
E-Cig					
	Age	−0.332	0.007	−0.347	−0.318	<0.001
	FIPR x Race (Black)	0.066	0.015	0.037	0.094	<0.001
	Employed	−0.017	0.008	−0.033	−0.001	0.035
	Black	−0.162	0.015	−0.191	−0.132	<0.001
	Male	0.051	0.007	0.038	0.064	<0.001
	Education	−0.072	0.007	−0.086	−0.057	<0.001
	FIPR	−0.065	0.009	−0.081	−0.048	<0.001
	Married	−0.088	0.007	−0.102	−0.074	<0.001
	Intercept	1.913	0.033	1.848	1.978	<0.001
						
Depression					
	Age	−0.088	0.008	−0.104	−0.072	<0.001
	FIPR x Race (Black)	0.066	0.015	0.036	0.097	<0.001
	Employed	−0.074	0.009	−0.091	−0.057	<0.001
	Black	−0.099	0.016	−0.130	−0.068	<0.001
	Male	−0.025	0.007	−0.039	−0.012	<0.001
	Education	−0.025	0.008	−0.040	−0.009	0.002
	FIPR	−0.105	0.009	−0.123	−0.087	<0.001
	Married	−0.028	0.007	−0.043	−0.014	<0.001
	Intercept	0.915	0.037	0.843	0.987	<0.001

## 4. Discussion

This study aimed to elucidate the disparities in the impact of the FIPR—a widely used relative measure of SES—on various health outcomes between Black and White adults. Focusing on depression, obesity, and e-cigarette use, the research sought to provide a comprehensive overview of potential discrepancies in the health benefits afforded by socioeconomic advancements across racial lines.

Williams has extensively discussed the impact of place and social determinants of health (SDOH) as significant contributors to racial health disparities [19]–[22]. He argued that SES, racism, and discrimination play crucial roles in these disparities within Black communities. In his work, Link emphasized the role of structural racism as a fundamental cause of poor health outcomes in Black populations [23]–[25]. Similarly, Krieger [26]–[29], Gee [21],[30]–[33], and Assari [34],[35] have all delved into the consequences of structural and systemic racism, shedding light on its profound impact on health disparities. Furthermore, Braveman [15],[16],[36] and Kaufman [37],[38] have contributed to this discourse by highlighting how SES metrics are not directly comparable across different racial and ethnic groups, further complicating the understanding of health inequalities. Our own research has further demonstrated that SES may have a lesser effect on the health outcomes of Black individuals compared to White individuals, highlighting the pervasive influence of structural racism and discrimination [39]–[41].

This research represents the first instance of demonstrating the diminished returns associated with a relative income measure, specifically the “FIPR” [42]. This ratio, also known as the poverty ratio or the income-to-needs ratio, includes a set of widely used economic terms that quantify the income level of an individual or family relative to the poverty threshold as defined by governmental or other authoritative entities [43],[44]. Acting as indicators of economic self-sufficiency, they reflect the SES of entire families, not just individual income levels, in comparison to the poverty threshold [45],[46]. These measures indicate the extent to which an individual's or family's income exceeds the poverty line, marking a degree of either financial independence or self-sufficiency. They offer an assessment of the relative economic standing, with a higher score indicating a higher SES. Essentially, these measures focus on the relationship between income levels and the poverty line, emphasizing the comparison of household income to the established needs for various household sizes and compositions [47],[48].

Our current analysis of the 2022 National Health Interview Survey data has unveiled significant interactions between the FIPR and race, indicating that SES distinctly influences various health outcomes. These findings reinforce the existing literature, consistently highlighting a nuanced intersection of SES and race in health disparities [49],[50]. This aligns with Navarro's perspective on race and SES [51]–[53], rather than either race or SES, as a cause of health disparities, and emphasized the importance of what SES can provide, not just the availability of SES resources for a particular group. When SES resources are available but not accessible, and if a high SES in adulthood is countered by low SES tolls during earlier years [54]–[56], the implications are profound.

Similarly, our analysis indicated that a higher SES, as measured by FIPR, is linked to lower rates of depression. Yet, for Black individuals, the protective effects of a higher SES against depression were significantly attenuated. This is consistent with previous research which indicated that high SES Black males were at a higher risk of depression [57].

Our findings regarding the relationship between the FIPR and obesity mirror the existing literature, suggesting that although SES inversely correlates with obesity prevalence [58]–[62], Black individuals experience a diminished protective effect of a higher SES against obesity [63]. This pattern has been consistently reported in earlier research, highlighting the role of race in influencing health outcomes beyond economic factors.

In line with past studies [64]–[67], our research demonstrated that a higher FIPR is associated with reduced tobacco use. However, this association exhibited racial disparities, with Black individuals not experiencing the same degree of reduction in tobacco use as their White counterparts. This finding corroborates previous research [68] that identified the diminished returns of socioeconomic improvements on the health behaviors among Black populations across various age groups. Reflecting trends observed in the broader literature, our study found an inverse relationship between the FIPR and e-cigarette use. Yet, the protective effect of a higher SES on e-cigarette use was less evident among Black individuals, which confirmed the previously reported pattern. Similarly, our analysis supported the established literature by showing that an increased FIPR generally led to lower instances of alcohol binge drinking. However, Black individuals did not benefit from this protective effect to the same extent as White individuals [69], reinforcing findings from previous studies that pointed to the complex interplay of race and socioeconomic status in influencing alcohol-related behaviors.

These results across health outcomes—depression, obesity, and e-cigarette use—aligned with the existing body of literature. They underscored the pervasive nature of racial disparities in the health benefits conferred by socioeconomic status improvements, highlighting the critical need for targeted interventions to address the unique challenges faced by Black individuals in realizing the full health potential of their socioeconomic gains [69].

The concept of the MDRs [69] provided a crucial framework to interpret our findings. Specifically, our study demonstrated that various SES indicators, such as the FIPR, did not uniformly translate into health benefits across racial groups. For Black individuals, achieving a higher SES did not result in the same degree of improvement in health outcomes as observed among their White counterparts. This discrepancy underscores the systemic barriers and ongoing effects of racism that disproportionately affect Black communities, limiting their ability to leverage socioeconomic gains for health improvement [69].

### 4.1. Implications

The implications of our findings are multifaceted, underscoring the urgent need for targeted policy interventions and healthcare strategies. Addressing the root causes of the MDRs requires a comprehensive approach that acknowledges and tackles the structural inequalities embedded within our society. Healthcare providers, policymakers, and community leaders must collaboratively work to create environments that enable Black individuals to fully realize the health benefits of socioeconomic advancements. This includes, but is not limited to, enhancing their access to quality healthcare, improving their living conditions, and ensuring equitable employment opportunities.

### 4.2. Limitations

While our study provided insightful findings, it is not without limitations. The cross-sectional design restricts our ability to infer causality between the FIPR and the health outcomes. Additionally, the reliance on self-reported measures may introduce bias. Furthermore, our findings may not be generalizable to all racial and ethnic minority groups, given the specific focus on Black and White adults.

### 4.3. Future research

Future research should aim to explore the mechanisms underlying the observed MDRs, with a particular focus on longitudinal studies that can better elucidate the causal relationships. Additionally, further investigation into the role of other SES indicators and their interaction with race and health is warranted. Understanding the complex interplay between socioeconomic factors, race, and health outcomes is crucial to develop effective interventions.

## 5. Conclusions

Our study highlighted the persistent racial disparities in health that stemmed from the diminished returns of socioeconomic status among Black individuals. By shedding light on these critical issues, we underscored the need for concerted efforts to address the underlying causes of health inequities. Achieving true health equity requires systemic changes that go beyond the individual socioeconomic advancements, calling for a collective commitment to dismantling the structural barriers that perpetuate disparities.

## Use of AI tools declaration

The authors declare they have not used Artificial Intelligence (AI) tools in the creation of this article.
